# Three case reports of post immunization and post viral Bullous Pemphigoid: looking for the right trigger

**DOI:** 10.1186/s12887-017-0813-0

**Published:** 2017-02-23

**Authors:** Luca Baroero, Paola Coppo, Laura Bertolino, Stefano Maccario, Francesco Savino

**Affiliations:** 1 Dipartimento di Pediatria 1, Ospedale Infantile Regina Margherita, Regina Margherita Children’s Hospital, Citta’ della Salute e della Scienza di Torino, Piazza Polonia 94, 10126 Turin, Italy; 2 Dipartimento di scienze mediche, Università degli studi di Torino, Torino, Italy; 3 Pediatric Department, Chelsea and Westminster Hospital, London, UK

**Keywords:** Case report, Infant, Bullous Pemphigoid, Drug therapy, Vaccination

## Abstract

**Background:**

Bullous pemphigoid (BP) is a blistering skin disorder infrequent in infancy and rarely reported in medical literature.

**Case Presentation:**

Here we describe three cases of BP which were referred to our department in the last 15 years. Two of them developed an eruption of bullous lesions just a few days after vaccination for diphtheria, tetanus, pertussis, poliomyelitis, hepatitis B and Haemophilus influenzae B. The third patient developed the same blistering lesions shortly after herpetic stomatitis. In all three cases, clinical diagnosis was confirmed by histological examination which showed subepidermal bullae with a dermal inflammatory infiltrate, and direct immunofluorescence of perilesional skin showed linear IgG and C3 deposits along the basement membrane zone. Immunoblot assay was positive for BP antigen 180. Treatment with oral prednisone was instituted and the lesions resolved in two out of three patients; the third one was treated with an immunosuppressive agent (tacrolimus) and corticosteroid and subsequently with intravenous immunoglobulin and plasmapheresis, due to an underlying complex autoimmune disease.

**Conclusion:**

Although the mechanism of induction of BP is still unclear, the close relationship between trigger events (immunization or viral infection) and onset of the disease arises a possible association.

## Background

Bullous pemphigoid (BP) is an autoimmune blistering skin disorder associated with presence of tissue-bound and circulating IgG autoantibodies directed against hemidesmosomal proteins, called BP antigen 180 and BP antigen 230 [1]. Bullous pemphigoid usually affects the elderly and is rare in childhood and infancy. BP is diagnosed on the basis of clinical, histologic and immunologic findings [2, 3]. Among possible trigger factors of BP, immunization and viral infections are mentioned in literature. Some cases of BP have been reported soon after vaccine administration, although the immunological mechanism underneath is still unclear [4–6].

The clinical presentation of BP amongst children differs from that seen in adults, notably in terms of acral involvement with predominance of palmoplantar lesions, sparing the mucosa and genital area, in children aged less than 1 year. Unlike in adults, childhood BP has usually a good prognosis and resolves quite rapidly after initiation of treatment [7].

Although a clear trigger is not well established for BP, especially in infancy, a combination of multiple factors can be postulated. We present here 3 cases of children younger than 2 years who were referred to our Hospital in the last 15 years after developing BP related in time with a previous episode of vaccination or viral infection.

## Case Presentation

**Fig. 1:**
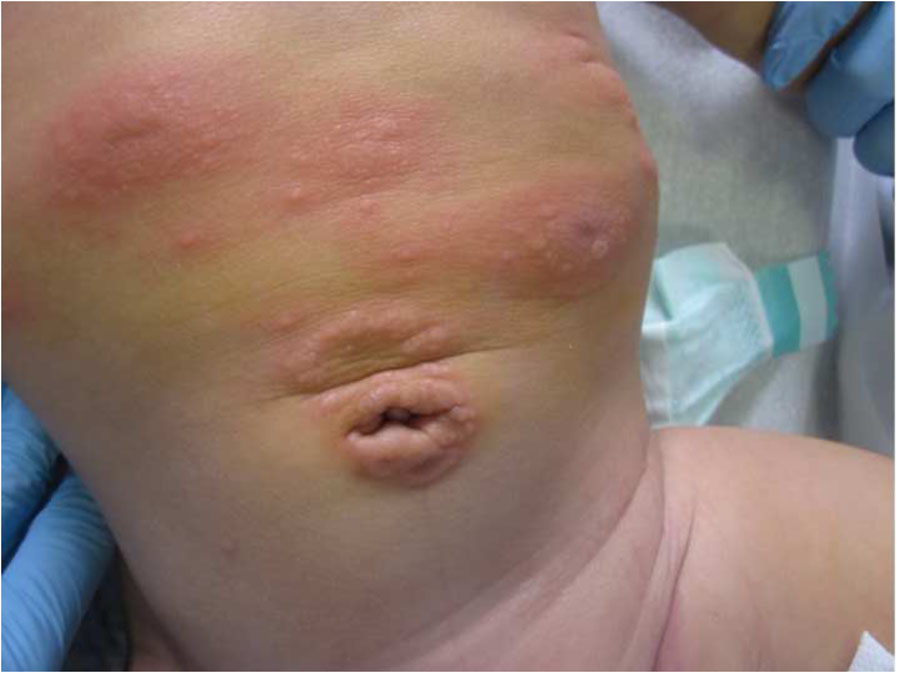
Patient 1: widespread small blisters on erythematous skin of the trunk and abdomen

**Fig. 2:**
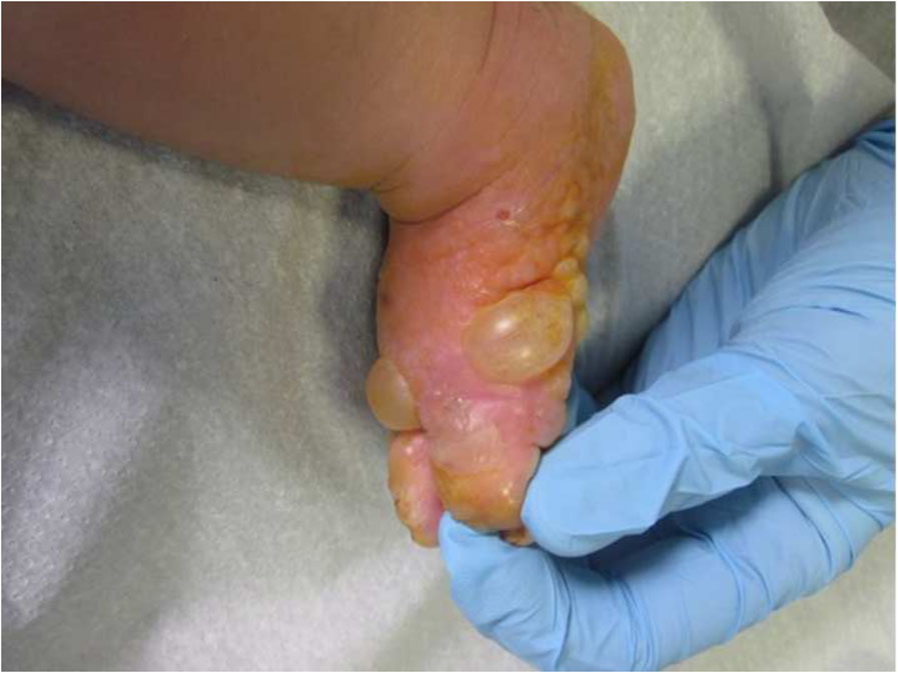
Patient 1: large vesicles and tense bullae with surrounding erythema located in feet, with palm and sole involvement

**Fig. 3:**
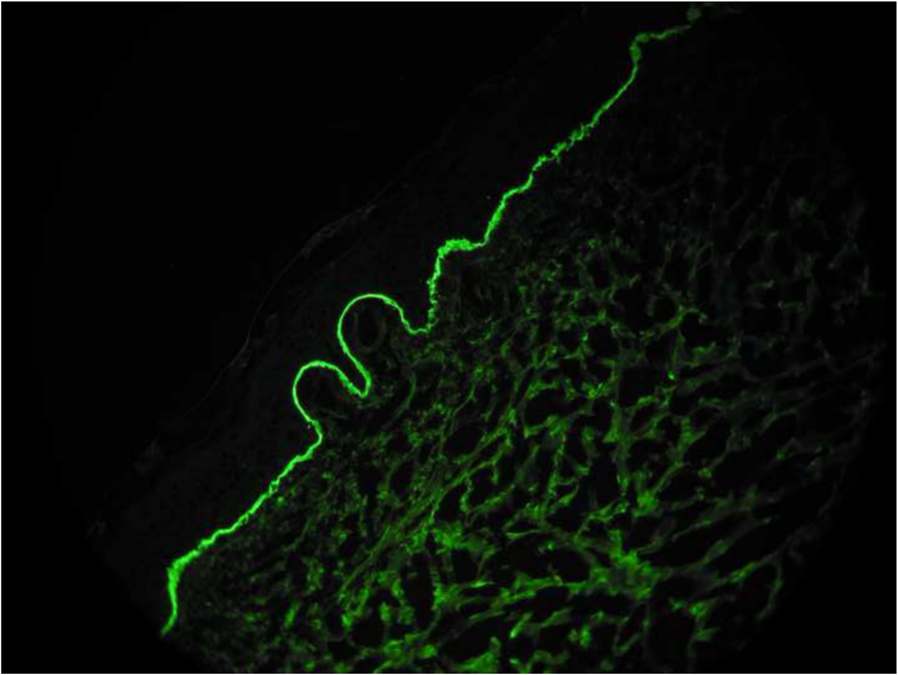
Patient 1: direct immunofluorescence showing linear deposition of IgG along the basement membrane zone

A previously healthy 3-month-old boy was referred to our Hospital with a 15-day history of a blistering eruption on his hands and feet. He received a first dose of combined vaccination against diphtheria, tetanus, pertussis, poliomyelitis, hepatitis B and Haemophilus influenzae B 2 days before the onset of the bullous rash. He had been previously treated at home with topical gentamicin and oral co-amoxiclavulante, without resolution of the skin eruption. There was no relevant family history for autoimmune or blistering disorders and no risk factors during pregnancy or delivery had been identified. Infant was breast-fed and growing normally. Clinically he presented with blistering lesions with a prevailing acral distribution: large vesicles and tense bullae with surrounding erythema were seen on the palms and soles, whereas widespread smaller blisters on erythematous skin could be noticed on the trunk and abdomen (Figs. 1 and 2). Mucous membranes were not involved and other systems’ examination was unremarkable. Observations were within normal limits and the patient was afebrile. Results of laboratory investigations showed that the patient had a mild eosinophilia (1.47 × 10^9^/L, 12% of WBC count); inflammatory markers and complement components were normal. Bacteriology analysis of the fluid inside blisters revealed no infections and blood virological tests were negative. A first biopsy for histologic study was taken from a recent vesicular lesion and showed subepidermal blister with a mixed superficial perivascular inflammatory infiltrate with abundant eosinophils. A second biopsy for direct immunofluorescence (DIF) was taken from uninvolved perilesional skin: the results of DIF showed linear deposition of immunoglobulin G (IgG, faint deposits) and complement component 3 (C3, intense deposits) along the basement membrane zone leading to the diagnosis of bullous pemphigoid (Fig. 3). The immunoblot assay was positive for BP antigen 180. Oral steroids have been started with prednisone at 1.5 mg/kg/day for ten days. Once the development of blisters was stopped and erythema had subsided, a careful tapering of prednisone was started, following an alternate day scheme. Considering the severity of the disease and the young age of infants, we started with a higher dose than suggested in guidelines [2]. For the whole duration of steroid treatment, the patient was subjected to a strict follow-up: therapy was well tolerated, with no adverse effects, as hypertension, weight gain, hyperglycemia or other blood test alterations. Prednisone was carefully tapered off over a 2-month period with no evidence of disease relapse and currently the patient is still in remission. Resumption of the vaccination schedule did not induce any recurrence of the disease.

A 17-month-old girl with a history of eczema and autoimmune enteropathy developed a blistering eruption on her hands and feet a few days after the second dose of hexavalent vaccination. Considering the autoimmune disorder affecting her gut, on the recommendation of gastroenterologists, she was treated with an immunosuppressive agent (tacrolimus) and corticosteroid; during a suspension of therapy for remission of gastrointestinal symptoms, she received the first dose of hexavalent vaccination at the age of 15 months with appearance of a single blister on the back of one hand 5 days later. At the age of 17 months 7 days after the second dose of vaccination, she developed a bullous rash on the limbs which subsequently spread to the whole body. Two punch biopsies were taken, one for the histologic examination and the other for DIF, and they led to the diagnosis of bullous pemphigoid. The girl was treated with oral prednisone 1 mg/kg/day; as the lesions did not improve the dose was increased to 2 mg/kg/day but still without benefit. Subsequently she received intravenous Ig and finally plasmapheresis (5 sessions) with full recovery within 6 months. The patient developed over the years an IPEX-like syndrome caused by deficiency of CD25 (IL2-RA), characterized by immunodeficiency and autoimmunity, which was genetically confirmed. She recently underwent a bone marrow transplant with success.

A 2-month-old girl with unremarkable family history for bullous diseases, developed an eruption of bullous lesions, on an erythematous base, confluent, located in both hands and feet, with palm and sole involvement, together with multiple ovaloid erythematous plaques, some with vesicles, on the abdomen. Ten days before she had been diagnosed with acute gingivostomatitis subsequently confirmed by PCR detection of HSV-1 DNA as herpetic stomatitis. At the beginning, to avoid bullous impetigo, the infant was managed with intravenous co-amoxiclavulanate. IgM antibody titer against HSV-1 was positive and suggestive of recent infection. A skin biopsy subsequently confirmed BP, showing sub-epidermal blisters. A second biopsy for direct immunofluorescence DIF showed linear deposits of IgG and C3 at the epidermal BMZ, confirming the diagnosis of bullous pemphigoid. Immunoblot assay was positive for BP antigen 180. The infant was managed with oral prednisone 1 mg/kg/day with rapid improvement, and she became free of blisters after 3 weeks of treatment. Follow-up to 6 months was good.

All parents of the three reported cases provided written informed consent to the inclusion of data concerning their infants in the manuscript in compliance with the Helsinki Declaration.

## Discussion

The reported cases are presentations of bullous pemphigoid, the most prevalent autoimmune blistering skin disease, presenting with tense blisters on erythematous skin, predominantly affecting elderly people and unusual in infancy. Bullous pemphigoid is usually a self-limiting disease with a clinical course that may last from months to years in adults. In childhood and infancy BP usually responds well to conventional treatments, with a good prognosis [8].

The etiopathogenesis of bullous pemphigoid is complex and during recent years much has been postulated regarding the trigger factors related to the development of this condition, as immunizations and viral infections [9–11]. None of our patients had a suggestive family history for blistering skin disease and specifically their mothers did not develop gestational pemphigoid during pregnancy.

Here we report the case of two infants who developed an eruption of bullous lesions just a few days after vaccination against diphtheria, tetanus, pertussis, poliomyelitis, hepatitis B and Haemophilus influenzae B, while the third patient after a viral infection by HSV-1. The latency period ranged between 2 and 10 days. A too short interval from immunization to onset of skin lesions could be considered an argument against the existence of a true relationship: since IgG production begins 10–14 days post-immunization, a 2–3 day latency period would generally be considered too short a time-frame for autoimmune manifestations characterized by IgG deposition to develop. Some authors anyway have suggested that certain vaccines may unmask subclinical BP by inducing a nonspecific immune reactivation in genetically predisposed infants more sensitive to the stimulus [8]. Others hypothesized that intrauterine transmitted maternal IgG antibodies might play a role [4] but a vertical transference of antibodies seems unlikely since in all the cases of PB reported in the literature where tests on mother’s serum were performed, circulating anti-BMZ antibodies were not found [11]. Moreover, according to recent studies, the trauma caused by the vaccine injection may led to Th17 cell activation with increased of IL-17 which is able to release pro-inflammatory cytokines and proteolytic enzymes, which may result in blister formation [12]. Finally, CD25 deficiency may be related to BP, since the lack of CD25+ cells has been observed in bullous pemphigoid lesions [13].

In literature some tens of cases of childhood BP have been reported, of which about 20 have been related to vaccine administration, but only a few occurred in infants younger than 6 months of age [6, 10, 14]. Anyway, the association of BP and vaccination could be entirely coincidental, given that vaccination in infants is a usual and daily practice in developed countries while cases of BP reported in infants are really limited. The high rate of vaccinations in the first year of life in contrast with the low number of reported cases of BP after vaccination makes it difficult to explain a causal relationship, even if described cases of recurrence after a new dose of vaccination seem to reinforce the hypothesis of a causal association [8].

Although the histopathological and immunological features of infantile BP are indistinguishable from those of childhood BP and adult BP, age-related differences in regional distribution of lesions were demonstrated. A recent study found that lesions are more likely located on the extremities during the first year of life [15]. For this reason, the clinical presentation of infantile BP seems to differ from that of childhood and adult BP, which are characterized by tense blisters predominantly appearing along folds in the skin on the lower abdomen, groin, upper thighs and arms. In our cases there was no correspondence between the location of the vaccine administration and the site of occurrence of the first lesions.

The diagnosis of BP in our three cases has been confirmed with DIF studies on perilesional skin, which showed linear deposits of IgG and/or C3 at the epidermal BMZ [16]. To perform the DIF, frozen sections fixed in acetone at a temperature of 4 °C were incubated with Ig fluorescein isothiocyanate in humid chamber (IgA,IgM,C3 diluite 1/10; IgG 1/20), then rinsed in PBS and covered with anti-fade mounting medium.

Laboratory investigations are nonspecific, while histopathologic analysis shows sub-epidermal blisters. Diagnostic findings for BP are listed in Table 1. In our cases indirect immunofluorescence and detection of circulating autoantibodies against PB antigens were not performed.

**Table 1:** Diagnostic findings for BP

Clinic	Blistering lesions on erythematous skin, with a prevailing acral distribution
Histology	Subepidermal blister with a mixed perivascular infammatory infiltrate
Direct immunofluorescence microscopy	Linear deposits of IgG and C3 along the basement membrane
Indirect immunofluorescence microscopy on salt-split-skin	BP antibodies deposited primarily at the epidermal side of the induced blister
ELISA	Presence of circulating antibodies against the 2 BP antigens (BP180 and BP230)

Regarding differential diagnosis, BP should be differentiated from other subepidermal diseases: most of all DIF is useful in distinguishing BP from epidermolysis bullosa acquisita, mucous membrane pemphigoid and linear IgA disease. Bullous lesions may also be caused by insect bites, burns, cellulitis and contact dermatitis. Viral and bacterial skin infections should be recognized and treated before starting immunosuppressive therapy [7, 16].

Treatment with oral prednisone was instituted and the lesions rapidly resolved in two out of three patients, with suppression of inflammation and blistering typically achieved in a period of a few weeks, after which the dose was gradually reduced; the third one was treated with an immunosuppressive agent (Tacrolimus) and corticosteroid and subsequently with intravenous immunoglobulin and plasmapheresis, due to a complex underlying autoimmune disease [7].

According to a Cochrane review by Kirtschig et al. oral corticosteroid drugs are the most common treatment regimens and starting doses of prednisolone of 0.75 mg/kg/day or less seem to be adequate to control disease and reduce the incidence and severity of adverse reactions [17].

Other treatments with reported benefit are potent topical steroids, azathioprine, mycophenolate mofetil, dapsone, methotrexate, cyclosporin, cyclophosphamide, plasma exchange, as well as erythromycin and tetracycline as monotherapy or with nicotinamide [7, 17]. There is a small number of case reports for the use of intravenous immunoglobulin (IVIg) [18]. Reports have also described successful therapy of BP patients with rituximab in treatment-refractory forms [19].

Since up to 40% of patients with BP on systemic corticosteroids develop severe infectious complications resulting in hospitalization or death [20] we administered a broad-spectrum antibiotic therapy to our 3 patients.

## Conclusion

In this article we reported two infants who developed an eruption of bullous lesions just a few days after vaccination against diphtheria, tetanus, pertussis, poliomyelitis, hepatitis B and Haemophilus influenzae B, while the third patient showed the same lesions after a viral infection by HSV-1. Although the mechanism of induction is unclear, the close relationship between trigger events and onset of the disease suggests that there may be an association. Treatment with oral prednisone was effective in achieving disease control in two out of three patients; the third one was treated with a combination of systemic corticosteroids and Tacrolimus and subsequently with intravenous immunoglobulin and plasmapheresis, due to a complex underlying autoimmune disease. BP is an uncommon autoimmune skin disorder in infancy, although recently some cases have been reported after vaccinations or viral infections [21, 22]. In most cases it shows prominent palmoplantar involvement and responds well to systemic steroid therapy, even if recognizing it promptly is important to establish appropriate treatment and prevent infectious complications which may be common and severe. More research will in fact be necessary to refine and further elaborate our knowledge on right trigger events of BP in infants.

## Abbreviations

BMZ: Basement membrane zone
BP: Bullous pemphigoid
C3: Complement component 3
DIF: Direct immunofluorescence
HSV-1: Herpes simplex virus type 1
IgG: Immunoglobulin G
IgM: Immunoglobulin M
PCR: Polymerase chain reaction
